# Proliferative glomerulonephritis with monoclonal immunoglobulin deposits (PGNMID): three Case Reports and systematic review

**DOI:** 10.3389/fmed.2025.1637435

**Published:** 2025-09-15

**Authors:** Quanying Ji, Shuyuan Yu, Xia Zhou, Zhenhua Wang, Xiaoqing Guo, Yuping Zhong

**Affiliations:** ^1^School of Clinical Medicine, Shandong Second Medical University, Weifang, Shandong, China; ^2^Department of Hematology, Qingdao Municipal Hospital, Qingdao, Shandong, China

**Keywords:** proliferative glomerulonephritis with monoclonal immunoglobulin deposits, glomerulonephritis, daratumumab, case report, proteinuria

## Abstract

**Objective:**

This study aimed to investigate the clinical characteristics and personalized treatment strategies for proliferative glomerulonephritis with monoclonal immunoglobulin deposits (PGNMID).

**Methods:**

A retrospective analysis was conducted on the clinical features, pathological characteristics, and treatment regimens of three PGNMID patients.

**Conclusion:**

Proliferative glomerulonephritis with monoclonal immunoglobulin deposits patients commonly present with proteinuria, hematuria, and renal insufficiency. Pathologically, light microscopy predominantly reveals a membranoproliferative glomerulonephritis (MPGN) pattern, with IgG3κ being the most prevalent immunohistochemical subtype. Current guidelines recommend the BCD regimen (bortezomib, cyclophosphamide, dexamethasone) as first-line therapy. Daratumumab may be a safe and effective therapeutic option for PGNMID; however, clinical decision-making should comprehensively consider patient age, renal function status, treatment tolerance, and other factors.

## Introduction

Proliferative glomerulonephritis with monoclonal immunoglobulin deposits (PGNMID) is a rare renal disease belonging to the spectrum of monoclonal gammopathy of renal significance (MGRS). Its hallmark feature involves glomerular deposition of monoclonal immunoglobulin (M-protein), which mediates immune complex formation and subsequent tissue injury (1, 2). Patients typically present clinically with proteinuria, hematuria, and progressive renal impairment. Characteristic pathological findings include a membranoproliferative glomerulonephritis (MPGN) pattern on light microscopy, with immunofluorescence demonstrating monotypic immunoglobulin deposits (predominantly a single IgG subclass with restricted light chain expression). The IgG3κ subtype is most frequent (approximately 50% of cases), followed by IgG3λ (approximately 15%) and other IgG subclasses. Electron microscopy reveals electron-dense deposits in the mesangium, subendothelial, or subepithelial spaces (3, 4). Notably, although PGNMID is classified within the MGRS spectrum, M-protein is undetectable in the serum or urine of approximately 70% of patients (5), and pathogenic peripheral plasma cell clones are identified in only about 30% of cases (6, 7). Therefore, renal biopsy demonstrating monotypic light chain restriction by immunofluorescence has become the diagnostic gold standard.

Since the initial systematic description of the disease by Nasr et al. (3), clinical understanding of PGNMID has continued to evolve; however, significant challenges remain in its diagnosis and management. The primary goal of treatment is to suppress the production of pathogenic M-protein in order to slow the progression of renal impairment. Current international guidelines and literature recommend an individualized treatment strategy based on disease activity and clonality characteristics (8, 9): (1) Risk-adapted therapy: For patients with high disease activity or high-risk features (e.g., proteinuria > 1 g/day or rapidly declining renal function), chemotherapeutic regimens targeting clonal precursor cells are recommended. In contrast, patients with low disease activity and stable renal function (e.g., proteinuria < 1 g/day) may be managed conservatively with renin-angiotensin system inhibitors under close monitoring. (2) Clone-directed targeted therapy: Treatment selection should be guided by the type of M-protein and its cellular origin. For IgG-type PGNMID: Pathogenic IgG is primarily secreted by long-lived plasma cells that have undergone terminal differentiation and do not express CD20. Therapy should therefore include agents directly targeting plasma cells, such as proteasome inhibitor-based regimens containing bortezomib (e.g., the BCD regimen), or anti-plasma cell agents targeting CD38 (e.g., daratumumab). For IgM-type PGNMID: Pathogenic IgM is typically produced by CD20-positive precursor cells, such as plasmablasts or activated B cells. Thus, anti-CD20 monoclonal antibodies (e.g., rituximab) represent a more suitable treatment option.

Against this background, the present study reviews three illustrative cases of PGNMID with distinct clinical presentations and treatment pathways: one managed conservatively, another treated with a bortezomib-based regimen, and a third achieving sustained remission with daratumumab after relapsing following initial chemotherapy. By integrating these case-based observations with current literature, we aim to explore individualized therapeutic strategies for PGNMID and to inform its clinical management.

## Case presentation

### Case 1

A 14-year-old male presented with “increased urinary foam for 3 days” without identifiable triggers. Outpatient urinalysis revealed urine protein 3+ and urine occult blood 3+. No previous special medical history. Physical examination showed stable vital signs and no significant abnormalities.

#### Laboratory investigations

Serum creatinine was 49.44 μmol/L, eGFR: 149.2 mL/ min/1.73 m^2^; urinalysis revealed protein 3+ and occult blood 2+, with a 24-h urine protein quantification of 1,341.63 mg. Anti-myeloperoxidase antibody levels were slightly elevated at 22.58 RU/mL. Urinary kappa light chains measured 10.2 mg/L (mild elevation), while lambda light chains and the κ/λ ratio remained within normal limits. Complement levels (C3 and C4) were unremarkable, and serum free light chain quantification and ratios showed no abnormalities. Serum and urine immunofixation electrophoresis detected no monoclonal bands. Viral serology for hepatitis B and C was negative. Bone marrow cytology and immunophenotyping revealed no clonal or morphological abnormalities, and autoantibody profiles (antinuclear antibodies and anti-phospholipid antibodies) were negative.

#### Renal biopsy diagnosis

**FIGURE 1:**
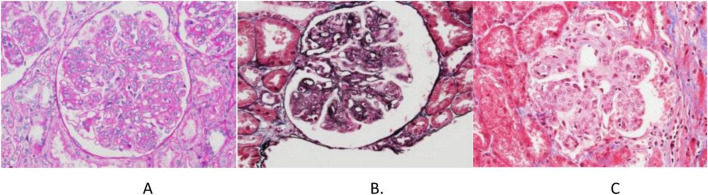
Renal biopsy findings. **(A)** Periodic acid–Schiff, magnification, ×400; **(B)** periodic-acid silver methenamine, magnification, ×400; **(C)** Masson’s trichrome staining, magnification, ×400.

**FIGURE 2:**
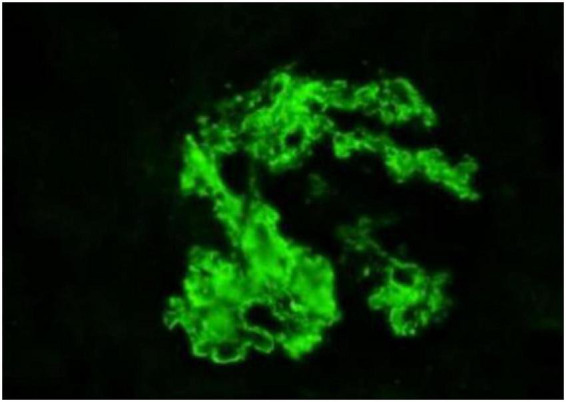
Immunofluorescence findings. Magnification, ×400.

The biopsy confirmed proliferative glomerulonephritis with monoclonal IgG deposits (IgG3-λ). Pathological findings: Light microscopy revealed 46 glomeruli with moderate to severe mesangial expansion, increased mesangial cells and matrix, segmental narrowing of capillary loops, and focal thickening/layering of the glomerular basement membrane. PASM-Masson staining identified scattered fuchsinophilic deposits in the mesangium and subendothelial regions. Mild acute tubular-interstitial injury with sparse mononuclear cell infiltration was observed, while arterial structures appeared unremarkable (Figure 1). Congo red staining was negative for amyloid. Immunofluorescence demonstrated granular deposits in 6 glomeruli: IgG (2+), IgM (1+), C3 (2+), C1q (2+), and λ light chain (2+) in the mesangium and capillary loops, with trace κ light chain positivity; IgA was negative. IgG subclass analysis showed IgG3 (2+) diffusely deposited in the mesangium and loops (Figure 2), while IgG1, IgG2, and IgG4 were negative. PLA2R staining on paraffin sections was negative. Electron microscopy revealed segmental glomerular basement membrane thickening, diffuse foot process effacement, mesangial matrix interposition, and electron-dense deposits in the subendothelial and mesangial regions without substructures. Immunoelectron microscopy confirmed predominant λ light chain deposition (significantly stronger than κ).

#### Treatment

Since March 2024, a conservative observation strategy has been adopted, and chemotherapy has not been initiated. The patient has received traditional Chinese medicine (TCM) treatment primarily focused on renal protection using Bailing capsules/tablets (*Cordyceps sinensis*), administered at a dosage of 2 g three times daily. This treatment has been continued for over 12 months. During this period, the lowest recorded proteinuria level decreased to 0.2 g/day, and renal function remained stable. The patient is currently maintained on continued Bailing capsule therapy and remains under ongoing follow-up.

### Case 2

A 60-year-old male presented with “head distension accompanied by nausea, vomiting, bilateral lower limb edema, and increased foamy urine for 1 day” without identifiable triggers. Past medical history included hypertension for over 30 years (maximum blood pressure 200/110 mmHg, controlled to 150/90 mmHg with oral antihypertensives) and saphenous vein varicosity surgery 15 years prior. Physical examination showed stable vital signs (blood pressure 160/80 mmHg), mild bilateral lower limb edema, and no other significant abnormalities.

#### Laboratory investigations revealed

Serum creatinine 113 μmol/L, eGFR: 66.1 mL/min/1.73 m^2^; urine protein 3+, urine occult blood+; 24 h urine protein quantification 4554 mg; complement C3 0.74 g/L, C4 0.23 g/L; serum free light chains: free κ 26.43 mg/L (free λ normal, κ/λ ratio normal); antinuclear antibody, anti-neutrophil cytoplasmic antibody, and anti-dsDNA antibody negative; bone marrow cytology: plasma cells accounted for 1.5%.

#### Renal biopsy diagnosis

Proliferative glomerulonephritis with monoclonal immunoglobulin deposits (IgG3-κ).

#### Pathological findings

Light microscopy revealed 10 glomeruli with moderate to severe diffuse hyperplasia of mesangial cells and matrix, accompanied by endothelial cell hyperplasia, lobulated capillary loops, luminal narrowing/occlusion, thickened basement membrane, mesangial interposition, and double-contour formation. Mesangial and subendothelial eosinophilic deposits were observed without significant parietal epithelial cell hyperplasia or crescent formation. Tubular epithelial vacuolar and granular degeneration, focal atrophy (∼15% area), interstitial focal inflammatory cell infiltration with fibrosis, and vascular lesions (segmental arteriolar hyalinosis and small arterial wall thickening with luminal narrowing) were noted. Immunofluorescence showed IgG+++, IgA−, IgM+, C3+++, IgG1−, IgG2−, IgG3+++, IgG4−, κ+++, and λ−, with diffuse granular deposits in the mesangial area and capillary loops. Electron microscopy demonstrated segmental glomerular basement membrane thickening, partial foot process effacement, mesangial interposition, electron-dense deposits in mesangial and subendothelial areas, and occasional subepithelial deposits.

#### Treatment

The patient initiated BCD therapy (bortezomib 1.3 mg/m^2^ on days 1, 8, 15, 22; cyclophosphamide 300 mg/m^2^ on days 1, 8, 15; dexamethasone 20 mg on days 1–2, 8–9, 15–16, 22–23) on January 16, 2024, and completed six cycles. Post-treatment evaluation revealed: serum creatinine 96 μmol/L, eGFR 73.7 mL/min/1.73 m^2^; urine protein 1+, urine occult blood trace-positive (±); 24-h urine protein excretion 266 mg; complement C3 0.73 g/L, complement C4 0.19 g/L; serum free light chain levels and κ/λ ratio were within normal limits. Serum creatinine and 24-h urine protein excretion normalized, consistent with a complete renal response. Clinically, edema significantly improved with no other subjective complaints reported. Following symptomatic improvement and normalization of laboratory parameters, the patient self-discontinued treatment. At the 3-month follow-up assessment after discontinuation, 24-h urine protein excretion had increased to 500 mg. Given the prior favorable treatment response, BCD therapy was therefore reinitiated. The patient continues to be followed up.

### Case 3

A 72-year-old male was admitted due to “proteinuria detected for over 6 years.” During a routine physical examination in 2018, urine protein 2+ was noted without bilateral lower limb edema; the patient did not seek further evaluation or treatment at that time. In February 2023, the patient developed facial and bilateral lower limb edema following exertion. Laboratory tests revealed serum albumin 31 g/L, serum creatinine 110 μmol/L, eGFR: 57.5 mL/min/1.73 m^2^, urine protein 3+, RBC 13.2/HP, and 24 h urine protein quantification 2970 mg.

#### Renal biopsy diagnosis

Proliferative glomerulonephritis with monoclonal gammopathy (κ type).

#### Pathological findings

Light microscopy revealed 10 glomeruli, including 1 ischemic sclerotic glomerulus (10%), with the remaining glomeruli exhibiting diffuse lesions characterized by moderate to severe cellular hyperplasia and mesangial matrix expansion, accompanied by extensive interposition and mild endothelial cell hyperplasia, along with hyaline degeneration. Congo red staining was negative. Immunofluorescence showed IgG+++, IgA+, IgM−, C3++, C1q−, FRA+, IgG1−, IgG2−, IgG3−, IgG4−, κ++, and λ± with petal-like deposits along capillary walls and granular deposits in the mesangial area. Electron microscopy demonstrated moderate to severe mesangial cell and matrix hyperplasia, segmental interposition, segmental double-contour formation in the basement membrane, electron-dense deposits in mesangial, subendothelial, and segmental subepithelial regions (some with uneven density), and extensive effacement of podocyte foot processes. Tubular findings included vacuolar degeneration of epithelial cells with increased lysosomes, partial loss of microvilli, and shed cellular debris in some tubular lumens. Interstitial changes comprised mild lymphomonocytic infiltration with collagen fiber hyperplasia. Bone marrow morphology and flow cytometry were unremarkable. Serum and urine immunofixation electrophoresis were negative. Serum free light chains: κ 51.40 mg/L, λ 28.19 mg/L (κ/λ ratio 3.14). The patient had a 1-year history of hypertension.

#### Treatment

##### Initial treatment

The patient initiated BCD therapy (bortezomib 1.3 mg/m^2^ on days 1, 8, 15, 22; cyclophosphamide 0.5 g on day 15; dexamethasone 20 mg on days 1–2, 8–9, 15–16, 22–23) in March 2023 and regularly completed eight cycles. Following treatment, the patient’s symptoms improved, and serum free light chain levels along with the κ/λ ratio normalized. Subsequently, the patient self-discontinued treatment. Two months after discontinuation, symptoms worsened, and reevaluation revealed significantly elevated serum creatinine and 24-h urine protein excretion (5671.76 mg). Bone marrow cytology examination showed plasma cells accounting for 0.5% of nucleated cells, and immunophenotyping revealed a plasma cell population comprising 0.33% with no immunophenotypic abnormalities detected.

##### Subsequent treatment

Given the short-term relapse following prior BCD therapy discontinuation, treatment was switched to the Dd regimen (daratumumab 800 mg per dose: administered weekly for 8 doses, then biweekly for 8 doses, with the regimen adjusted to monthly maintenance starting from the 17th dose; combined with dexamethasone 20 mg). A sustained complete response was achieved within 2 months of initiating the Dd regimen. The patient has now been followed up for over 1 year, remains asymptomatic, and maintains serum creatinine and 24-h urine protein excretion levels within normal ranges. Renal histopathological examination results and treatment of the three patients are summarized in Table 1.

**TABLE 1:** Renal histopathological examination results and treatment in three patients.

Parameters, unit	Case 1	Case 2	Case 3
Clinical manifestations	Sudden foamy urine	Headache, nausea, vomiting	Chronic proteinuria (>6 years)
24 h total protein, mg	1340	4554	2970
Serum creatinine, umol/L	49.94	113	110
Immunofixation electrophoresis	Negative	Negative	Negative
Plasma cell percentage in bone marrow aspiration light microscopy	0.5	1.5	0.5
Histological patterns	MPGN	MPGN	MPGN
Deposited proteins	IgG3-λ	IgG3-κ	Light chain κ
**Immunofluorescence**			
Pattern and site of deposition	Granular - mesangium, capillary loops	Granular - mesangium, capillary loops	Granular – mesangium petal-shaped pattern - capillary loops
Sites of electron-dense deposit deposition under electron microscopy	Mesangial area, subendothelial	Mesangial area, subendothelial, occasionally subepithelial	Mesangial area, subendothelial, segmental subepithelial
Treatment plan	Bailing capsule. No targeted therapy initiated.	BCD regimen	Switched to Dd regimen after BCD regimen recurrence
Treatment response	Urine protein fluctuates 0.2–1.0 g/d; normal renal function.	Relapsed after discontinuation; resumed BCD regimen with follow-up.	Sustained remission after regimen change.

## Discussion

### Analysis of treatment strategies and decision-making rationale

The three PGNMID cases in this series received distinct therapeutic regimens, reflecting a risk-adapted and clone-informed individualized treatment approach that aligns with the high heterogeneity of the disease.

Case 1 was a 14-year-old male with clinical features including young age, preserved renal function (eGFR 149.2 mL/min), relatively low-level proteinuria (<1.5 g/day), negative serum and urine immunofixation electrophoresis, no evidence of clonal plasma cells in bone marrow examination, and the absence of active pathological lesions such as crescents in renal tissue. Based on these low-risk and low disease-activity characteristics, a conservative management strategy was adopted. The patient received traditional Chinese medicine focused on renal protection, primarily consisting of Bailing capsules/tablets (active component: *Cordyceps sinensis*). Several meta-analyses (10, 11) have suggested that *Cordyceps*-based agents may mitigate nephritic inflammation and reduce proteinuria through immunomodulatory and anti-inflammatory effects. Follow-up showed a significant decrease in proteinuria with stable renal function. This case indicates that for strictly selected low-risk patients, active conservative management combined with close monitoring may avoid the toxic side effects associated with immediate chemotherapy. However, it must be clearly stated that the role of traditional Chinese medicine in the treatment of PGNMID remains exploratory and lacks support from high-level evidence. Its application requires caution, and it is essential to be aware that approximately 25% of patients may progress to end-stage renal disease within 3 years (4, 12).

Case 2 was a 60-year-old male who presented with high disease activity, including nephrotic-range proteinuria (4.55 g/day) and mild renal impairment (eGFR 66.1 mL/min). Accordingly, a bortezomib-based BCD regimen was selected for targeted treatment. The patient achieved complete remission, consistent with the known mechanism of proteasome inhibitors in eliminating plasma cells and suppressing M-protein secretion through inhibition of the NF-κB pathway, among other effects (13). Disease recurrence following self-discontinuation of therapy not only confirms the efficacy of the BCD regimen in inducing remission, but also indicates that certain patients may require extended consolidation or low-dose maintenance therapy to sustain remission.

Case 3 presented a more complex clinical course. This 72-year-old male initially responded to the BCD regimen; however, shortly after treatment was discontinued, the disease relapsed (within 2 months) with rapid progression to nephrotic-range proteinuria, suggesting a potentially resistant or high-risk disease phenotype. Renal biopsy immunofluorescence showed prominent IgG (+++) and κ light chain (++) deposits, whereas all IgG subclasses (IgG1–4) were negative and λ light chain expression was suppressed (±). This unusual staining pattern suggests that the glomerular deposits may not consist of intact IgG molecules. Instead, they are more likely composed of structurally abnormal immunoglobulins, such as pathogenic species made up of heavy-chain fragments (possibly lacking functional domains like CH2/CH3) associated with κ light chain (14). Treatment was switched to daratumumab (Dara), which has a distinct mechanism of action. Following the transition, the patient rapidly achieved and maintained deep remission. Unlike therapies targeting the secreted protein, Dara eliminates aberrant plasma cells through dual mechanisms–antibody-dependent cellular cytotoxicity (ADCC) and complement-dependent cytotoxicity (CDC) (15, 16). This result is consistent with a reported case of a 15-year-old patient with PGNMID who achieved resolution of proteinuria and normalized renal function after 6 months of Dara monotherapy (17), supporting daratumumab as an important therapeutic option for relapsed/refractory PGNMID.

### Key implications

Based on the experience from this case series and supporting literature, the following key implications emerge for the clinical management of PGNMID:

(1)    Treatment decisions should integrate both clonality and clinical disease activity. For patients with detectable serum M-protein or a confirmed clone, the goal should be hematologic complete response (CR). In seronegative patients, as exemplified in this cohort, the decision to initiate intensive treatment should be guided primarily by clinical disease activity, such as proteinuria > 3.5 g/day, progressive decline in renal function, or the presence of active pathological lesions.
(2)    Pathological findings may inform therapeutic choices. Immunofluorescence results on renal biopsy are not only critical for diagnosis–specific staining patterns may also reflect structural characteristics of the pathogenic immunoglobulin and provide insight into differential treatment responses, potentially guiding regimen adjustments (e.g., switching from a proteasome inhibitor to an anti-CD38 monoclonal antibody).
(3)    Treatment tolerance and relapse prevention warrant careful attention. Dose individualization and regimen modifications are essential in elderly patients or those with significant comorbidities. The relapse observed in Case 2 underscores that patients who achieve remission but have high-risk features–such as impaired baseline renal function, slow response to induction therapy, or previous relapse–may benefit from low-intensity maintenance strategies (e.g., reduced-dose or less frequent bortezomib, or extended intervals of anti-CD38 antibody administration) to mitigate the risk of recurrence.
(4)    Multidisciplinary team (MDT) collaboration is essential. Given the diagnostic challenges and frequent seronegativity in PGNMID, close collaboration among renal pathology, hematology, and laboratory medicine is crucial. Integrating pathological findings with clinical information enhances diagnostic accuracy and supports informed treatment decision-making.

### Pathogenic mechanisms and clinical implications

The core pathogenesis of PGNMID involves glomerular deposition of monoclonal immunoglobulin (M-protein) and subsequent complement activation, triggering inflammatory responses that result in glomerular injury.

Recent studies have proposed novel insights into its mechanisms: (1) Complexity of clonal origin: Approximately 70%–80% of PGNMID patients show no detectable M-protein or pathogenic B-cell clones in circulation, suggesting that deposited immunoglobulins may originate from occult small clones (below conventional detection thresholds) or oligoclonal activation (where some supposed “monoclonal” deposits may in fact represent convergent deposition of oligoclonal antibodies) (18). (2) Association with non-hematological disorders: A minority of cases are linked to viral infections (e.g., parvovirus B19, HCV) or solid tumors (e.g., lung cancer), supporting a possible paraneoplastic mechanism wherein tumor antigens drive a restricted antibody response within glomeruli. This is further corroborated by observed improvements in nephropathy following successful treatment of the associated tumor (19). Although complement activation remains central to disease progression, the precise cellular origin of the pathogenic M-protein has not yet been fully elucidated.

The unusual immunofluorescence pattern–strong IgG positivity with complete subclass negativity–observed in Case 3 may be attributed to the following mechanism: polyclonal anti-IgG antibodies, which recognize multiple epitopes, can bind to truncated heavy chains and produce a positive signal, whereas monoclonal antibodies against specific conformational epitopes of IgG subclasses fail to bind due to the absence of critical functional domains (e.g., CH2/CH3) (14). We speculate that the suboptimal response to the BCD regimen and early relapse in this case may be related to plasma cells producing abnormal immunoglobulins. Such cells, which may not engage in high-level synthesis of intact antibodies, could exhibit reduced sensitivity to bortezomib. In contrast, daratumumab directly targets CD38 on the plasma cell surface, independent of the structural properties of the secreted immunoglobulin, which may explain the sustained remission achieved after switching to Dara.

Therefore, when encountering similar immunofluorescence patterns (e.g., strong glomerular IgG staining with negativity for all subclasses) or patients resistant to first-line therapy, the possibility of aberrant immunoglobulin deposition should be considered. Such cases may be more effectively managed with treatment strategies directly targeting plasma cells (such as anti-CD38 monoclonal antibodies) rather than conventional regimens focused solely on secreted immunoglobulins. Although PGNMID typically features deposits of intact IgG, pan-negative IgG subclass staining suggests underlying structural variations and highlights the importance of further molecular pathological analysis in such cases (20).

### Future directions and limitations

Although targeted therapies have significantly improved outcomes in PGNMID, several unresolved issues remain: (1) optimal treatment duration and maintenance strategies for novel agents such as CD38-targeted antibodies; (2) methods for monitoring treatment response in patients lacking serological biomarkers; (3) the applicability of emerging therapies–including CAR-T cells and bispecific antibodies–in refractory cases; and (4) the need for prospective studies and rigorous evidence-based evaluation of traditional agents, such as those used in Case 1.

This study has several limitations. The small sample size (only three cases) and the relatively short follow-up period together limit the generalizability of our findings. Furthermore, the relatively short follow-up period precludes a comprehensive assessment of long-term outcomes, such as the sustained renal function, the risk of progression to end-stage kidney disease, and long-term treatment safety. Therefore, the observations presented here should be regarded as preliminary; further validation through larger, multicenter cohort studies is needed to confirm the efficacy of these individualized treatment strategies.

## Conclusion

In summary, this case series of three patients with distinct clinical features of PGNMID illustrates the application and preliminary outcomes of personalized treatment strategies based on risk stratification, disease activity, and renal biopsy characteristics. However, due to the limited sample size and short follow-up period, these findings should be considered exploratory. Optimized management of PGNMID remains dependent on validation through larger, well-designed prospective studies.

## Data Availability

The original contributions presented in the study are included in the article/supplementary material. Further inquiries can be directed to the corresponding authors.
